# Expanding the Use of HIPEC in Ovarian Cancer at Time of Interval Debulking Surgery to FIGO Stage IV and After 6 Cycles of Neoadjuvant Chemotherapy: A Prospective Analysis on Perioperative and Oncologic Outcomes

**DOI:** 10.1245/s10434-024-15042-0

**Published:** 2024-02-27

**Authors:** Valentina Ghirardi, Rita Trozzi, Francesca Romana Scanu, Diana Giannarelli, Francesco Santullo, Barbara Costantini, Angelica Naldini, Camilla Panico, Luciano Frassanito, Giovanni Scambia, Anna Fagotti

**Affiliations:** 1 Division of Gynecologic Oncology, Fondazione Policlinico Universitario A. Gemelli—IRCCS, Rome, Italy; 2 Universita’ Cattolica del Sacro Cuore, Rome, Italy; 3 Facility of Epidemiology and Biostatistics, G-STEP Generator, Fondazione Policlinico Universitario A. Gemelli, IRCCS, Rome, Italy; 4 Operational Unit of Peritoneum and Retroperitoneum Surgery, Fondazione Policlinico Universitario Agostino Gemelli IRCCS, Rome, Italy; 5 Department of Bioimaging, Radiation Oncology and Hematology, UOC of Radiologia Toracica e Cardiovascolare, Fondazione Policlinico Universitario A. Gemelli IRCSS, Rome, Italy; 6 Department of Emergency, Anesthesiological and Intensive Care Sciences, Fondazione Policlinico Universitario A. Gemelli, IRCCS, Rome, Italy

## Abstract

**Background:**

Randomized data on patients with FIGO stage III ovarian cancer receiving ≤ 3 cycles of neoadjuvant chemotherapy (NACT) showed that hyperthermic intraperitoneal chemotherapy (HIPEC) after interval debulking surgery (IDS) improved patient’s survival. We assessed the perioperative outcomes and PFS of FIGO stage IV and/or patients receiving up to 6 cycles of NACT undergoing IDS+HIPEC.

**Methods:**

Prospectively collected cases from January 1, 2019 to July 31, 2022 were included. Patients underwent HIPEC if: age ≥ 18 years but < 75 years, body mass index ≤ 35 kg/m^2^, ASA score ≤ 2, FIGO stage III/IV epithelial disease treated with up to 6 cycles of NACT, and residual disease < 2.5 mm.

**Results:**

A total of 205 patients were included. No difference was found in baseline characteristics between FIGO Stage III and IV patients, whereas rate of stable disease after NACT (*p* = 0.004), mean surgical complexity score at IDS (*p* = 0.001), and bowel resection rate (*p* = 0.046) were higher in patients undergoing delayed IDS. A lower rate of patients with at least one G3–G5 postoperative complications was observed in FIGO stage IV versus FIGO stage III disease (5.3% vs. 14.0%; *p* = 0.052). This difference was confirmed at multivariable analysis (odds ratio [OR] 0.24; 95% confidence interval [CI] 0.07–0.80; *p* = 0.02), whereas age, SCS, bowel resection, and number of cycles did not affect postoperative complications. No difference in PFS was identified neither between FIGO stage III and IV patients (*p* = 0.44), nor between 3 and 4 versus > 4 cycles of NACT (*p* = 0.85).

**Conclusions:**

Because of the absence of additional complications and positive survival outcomes, HIPEC administration can be considered in selected FIGO stage IV and patients receiving > 4 cycles of NACT.

In advanced ovarian cancer (AOC) diffuse carcinomatosis is the main responsible of the poor prognosis at late stage disease;^1^ therefore, the concept of targeting intraperitoneal disease either with surgery and/or with intraperitoneal chemotherapy has been pursued for a long time. The enhanced cytotoxic effect of hyperthermia to chemotherapy delivery has increased the interest toward hyperthermic intraperitoneal chemotherapy (HIPEC).^2^ Some promising survival results in a selected population who received HIPEC are coming from randomized data.^3^ At present, NCCN guidelines include HIPEC as a treatment option only in case of interval debulking surgery (IDS), where a survival advantage and not increased morbidity rate are supported by enough robust evidence.^3–5^ However, survival data appear to be limited to patients with FIGO stage III disease receiving 3 cycles of neoadjuvant chemotherapy (NACT).^3,6^

This study was designed to assess the effect of HIPEC at IDS in a series of cases including either FIGO stage IV and/or patients receiving up to 6 cycles of NACT.^6^ Outcomes were considered both perioperative morbidity and progression-free survival (PFS).

## Materials and Methods

From the publication of OVHIPEC-1 trial results in November 2018, HIPEC at the time of IDS has been introduced as standard clinical practice at Fondazione Policlinico Universitario Agostino Gemelli IRCCS.^3^ Since then and after receiving institutional review board approval from Fondazione Policlinico Universitario Agostino Gemelli IRCCS (No CICOG-04-03-19/5), data on patients undergoing interval surgery and HIPEC have been prospectively collected. We included in the current analysis patients receiving HIPEC from the inception to July 31, 2022.

Overall, patients were considered suitable to receive HIPEC after IDS if the following criteria were met: age ≥ 18 years but < 75 years, body mass index (BMI) ≤ 35 kg/m^2^, preoperative American Society of Anesthesiologists (ASA) score ≤ 2, at least a FIGO stage III epithelial ovarian cancer previously treated with up to 6 cycles of NACT with or without Bevacizumab addiction, a residual disease (RD) at the end of cytoreduction not greater than 2.5 mm.^6^

In addition, specific inclusion criteria for stage IV patients were: (1) complete response in parenchymal/distant metastases; (2) residual parenchymal/distant disease amenable to surgical resection or addressable with SBRT.

**Fig. 1:**
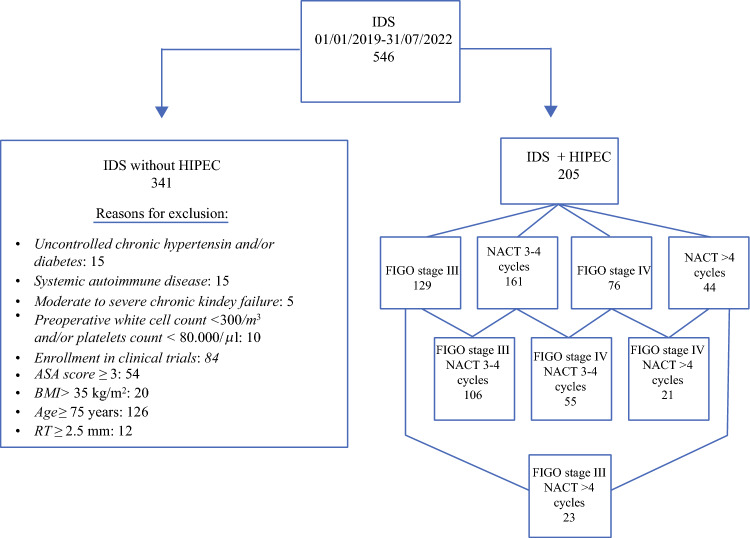
Flowchart of the study population

Exclusion criteria were as follows: uncontrolled chronic hypertension and/or diabetes; systemic autoimmune disease; moderate-to-severe chronic kidney failure (eGFR < 45–59 mL/min/1.73 m^2^), preoperative white cell count and platelets count < 3000/m^3^ and < 80,000/μl, respectively. Ultimately, enrollment in other clinical trials was considered an exclusion criterion for HIPEC administration. A flowchart of the study population with inclusion and exclusion criteria is depicted in Fig. 1.

In the study population, decision for NACT was made on a combination of whole body computed tomography (CT) findings, laparoscopic assessment of tumor load according to Fagotti score,^7^ and patient’s overall performance status through multidisciplinary tumor board (MDT) discussion. In selected cases deemed unfit for surgery, ultrasound guided biopsy was performed to achieve final histology. In every case, at the time of diagnostic laparoscopy, a 12-mm laparoscopic camera was placed at the level of the umbilicus and one or two 5-mm, additional trocars were placed along the midline of abdominal wall and in left iliac fossa, if needed. At the end of abdominal cavity exploration, to minimize the risk of port site metastases, pneumoperitoneum and ascites (whenever present) were both evacuated before trocars removal. For the same reason,all specimens were retrieved through an endobag.^8^

The number of NACT cycles was prolonged in case of poor response after three cycles, patients still unfit for surgery, or for a combination of highly complex surgery in frail woman. In some patients, the choice to perform NACT and number of cycles was taken in other institutions, and women were referred just for IDS.

Preoperatively, patients were admitted to hospital the day of surgery in keeping with Enhanced Recovery program.^9^ All patients underwent IDS through midline laparotomy after a diagnostic laparoscopy assessment to confirm patient’s operability with the primary goal to receive no gross residual disease (NGR).^10^ The only patients who received IDS through minimally invasive surgery (MIS) were those enrolled in the LANCE trial (NCT04575935) and were randomized accordingly.^11^

All surgical procedures were performed by gynecological oncologist. The assistance of other surgical specialties (e.g., epatobiliary surgeons or vascular surgeons) was requested in selected cases depending on preoperative and/or intraoperative findings.

Overall, reasons to perform stoma diversion included multiple bowel resection, level of rectal resection, vascularization, and tension of the anastomosis. The completeness of cytoreduction (CC) was determined by Sugarbaker’s criteria.^12^

If CC-0 or CC-1 was achieved at end of debulking procedure, HIPEC with cisplatin 100 mg/m^2^ at the temperature of 41 °C for 90 min was administered with closed technique. Right before HIPEC infusion, 9 g/m^2^ in 200 ml of saline of sodium thiosulfate was infused inytravenusly, followed by a continuous infusion of 12 g/m^2^ in 1000 ml of saline for 6 h after surgery.

During the postoperative period, a daily fluid balance was kept and a check of creatinine level every other day up to fifth day after surgery was performed, either in hospital or in the outpatient setting. In case of fluid retention, 20 mg of intravenous furosemide was administered.

Intraoperative complications were graded by using Common Terminology Criteria for Adverse Events (version 2.0), early postoperative complications (within 30 days from surgery) and 90 days postoperative complications were graded according to Clavien-Dindo classification.^13,14^ The time between the date of the IDS and the first adjuvant chemotherapy cycle was classified as time to start adjuvant chemotherapy.

### Statistical Analysis

This prospective study included consecutive patients undergoing IDS and HIPEC. Categorical variables were reported as absolute counts and percentages while quantitative items were summarized as median and range. Association between categorical factors was assessed by the Chi-square test, and differences in quantitative variables were evaluated with the Mann-Whitney nonparametric test. A logistic regression model was implemented to test the association between postoperative complications (any grade and G3-G5) and the number of NACT cycles, FIGO stage, patient’s age, SCS, and bowel resection.^6^

Survival times were estimated with the Kaplan-Meier method and differences between curves were assessed using the log-rank test. Differences were considered significant at a level of < 0.05. Progression-free survival was calculated from the date of IDS to the date of first recurrence, last follow-up, or death, whichever occurred first, or censored at the date of last follow-up. All analysis were performed with the software IBM-SPSS Statistics for Windows v.28.0 (Armonk, NY).

## Results

From January 2019 to July 2022, a total of 205 patients received IDS and HIPEC in our institution. Five (2.4%) of them were tretated through MIS as per randomization within the LANCE trial.^11^ No case of surgical resection/locoregional treatment for extra-abdominal metastatic disease sites was described in addition to main surgical procedure in the analyzed population.

Patients’ features are depicted in Tables 1 and 2. Seventy-six (37.1%) women were classified as FIGO stage IV ovarian cancer at diagnosis, of which 23 (11.2%) and 53 (25.9%) were staged as IVA and IVB respectively.^6^ Forty-four (21.5%) patients received > 4 cycles of NACT, of which 21 (47.7%) accounted for FIGO stage IV disease.^6^ Most of the patients had RT = 0 at the end of IDS (92.2%), an intermediate Surgical Complexity Score (SCS) (37.1%), and did not undergo bowel resection (58.5%). Among patients who had bowel resection, the rate of protective oostomy was 13.7%.

**Table 1:** Patients’ characteristics according to stage of disease

Characteristics	All cases *N* = 205	FIGO stage III *N* = 129 (62.9%)	FIGO stage IV *N* = 76 (37.1%)	*P* value
Age, median (range)	59 (28–74)	59 (28–73)	59 (37–74)	0.42
BMI,median (range)	24.1 (16.3–37.2)	23.8 (16.3–37.2)	25.0 (16.4–34.2)	0.21
FIGO stage				0.099
IIIC	129 (62.9)	129 (62.9)		
IVA	23 (11.2)		23 (11.2)	
IVB	53 (25.9)		53 (25.9)	
BRCA status				0.557
BRCA wild type	119 (58.0)	76 (58.9)	43 (56.6)	
BRCA1	50 (24.4)	30 (23.3)	20 (26.3)	
BRCA 2	29 (14.1)	18 (14.0)	11 (14.5)	
BRCA 1 and 2	1 (0.5)	–	1 (1.3)	
Missing	6 (2.9)	5 (3.9)	1 (1.3)	
NACT cycles, median (range)	3 (2–6)	3 (3–6)	3 (2–6)	0.25
Chemotherapy regimen				0.50
Carboplatin only	1 (0.5)	1 (0.8)	0	
Carboplatin-paclitaxel	171 (83.4)	109 (84.5)	62 (81.6)	
Carboplatin-paclitaxel +beva	33 (16.1)	19 (14.7)	14 (18.4)	
Chemotherapy response (RECIST)				0.99
Complete	1 (0.5)	1 (0.8)	0	
Partial	196 (95.6)	123 (95.3)	73 (96.1)	
Stable disease	8 (3.9)	5 (3.9)	3 (3.9)	
PI at IDS, median (range)	0 (0-6)	0 (0–6)	0 (0–6)	0.71
Residual disease at IDS^§^				
CC-0	189 (92.2)	117 (90.7)	72 (94.7)	
CC-1	16 (7.8)	12 (9.3)	4 (5.3)	0.30
SCS				0.86
Low	64 (31.2)	42 (32.6)	22 (28.9)	
Medium	76 (37.1)	47 (36.4)	29 (38.2)	
High	65 (31.7)	40 (31.0)	25 (32.9)	
Bowel resection				0.87
No	120 (58.6)	76 (58.9)	44 (57.9)	
Small-bowel resection	8 (3.9)	5 (3.9)	3 (3.9)	
Large-bowel resection	65 (31.7)	39 (30.2)	26 (34.3)	
> 1 resection	12 (5.8)	9 (7.0)	3 (3.9)	
Stoma formation				0.87
No	177 (86.3)	111 (86.0)	66 (86.9)	
Yes	28 (13.7)	18 (14.0)	10 (13.1)	
Ileostomy	23 (82.1)	15 (83.3)	8 (80)	0.82
Colostomy	5 (17.9)	3 (16.7)	2 (20)	
Estimated blood loss, median (range) ml	300 (50–2500)	300 (0–1500)	250 (50–2500)	0.10
Operating time, median (range)^a^	439 (50–840)	450 (175–840)	430 (255–740)	0.25
ICU admission				0.66
No	120 (58.5)	74 (57.4)	46 (60.5)	
Yes	85 (41.5)	55 (42.6)	30 (39.5)	
ICU stay, median (range) days	1 (1–4)	1 (1–4)	1 (1-2)	0.15
Length of stay, median (range) days	7 (3–36)	7 (3–36)	6 (3-32)	0.29
Time to start adjuvant chemotherapy, median (range)	36 (25–65)	35 (25–65)	38 (28-60)	0.48
Adjuvant chemotherapy regimen				0.64
Carboplatinum-paclitaxel	185 (90.2)	113 (87.6)	72 (94.8)	
Other	8 (3.9)	6 (4.6)	2 (2.6)	
No adjuvant	12 (5.9)	10 (7.8)	2 (2.6)	
ACT cycles, median (range)	3 (1–4)	3 (1-4)	3 (1-4)	0.40
Maintenance therapy				0.97
Yes	168 (81.9)	105 (81.4)	63 (82.9)	
No	35 (17.1)	22 (17.0)	13 (17.1)	
Missing	2 (1.0)	2 (1.6)	0	
Type of maintenance				
Bevacizumab	60 (29.3)	37 (28.7)	23 (30.3)	0.81
PARPi	109 (53.2)	68 (52.7)	41 (53.9)	0.87
Creatinine serum level Day 3, median (range)	0.60 (0.04–1.15)	0.60 (0.04–1.15)	0.60 (0.04–0.90)	0.70

*BMI* Body mass index; *PIV* Predictive index value; *NACT* Neoadjuvant chemotherapy; *IDS* Interval debulking surgery; *CC* Completeness of cytoreduction score; *SCS* Surgical complexity score; *ICU* Intensive care unit; *ACT* Adjuvant chemotherapy

^a^Including HIPEC perfusion time

**Table 2:** Patients’ characteristics according to number of NACT cycles

Characteristics	All cases *N* = 205	NACT 3–4 cycles *N* = 161 (78.5%)	NACT > 4 cycles *N* = 44 (21.5%)	*P* value
Age (years), median (range)	59 (28–74)	59 (28–74)	58 (41–72)	0.97
BMI, median (range)	24.1 (16.3–37.2)	24.0 (16.3–37.2)	25.2 (19.2–32.9)	0.44
FIGO stage				0.07
IIIC	129 (62.9)	106(65.8)	23 (52.3)	
IVA	23 (11.2)	14 (8.7)	9 (20.5)	
IVB	53 (25.9)	41 (25.5)	12 (27.2)	
BRCA status				0.186
BRCA wild type	119 (58.0)	91(56.5)	28 (63.6)	
BRCA1	50 (24.4)	39 (24.2)	11 (25.0)	
BRCA2	29 (14.1)	26 (16.1)	3 (6.8)	
BRCA1 and 2	1(0.5)	–	1 (2.3)	
Missing	6 (2.9)	5 (3.1)	1 (2.3)	
NACT cycles, median (range)	3 (2–6)			
Chemotherapy regimen				**0.006**
Carboplatin only	1 (0.5)	1 (0.6)	0	
Carboplatin-paclitaxel	171 (83.4)	140 (87.0)	31 (70.4)	
Carboplatin-paclitaxel +beva	33 (16.1)	20 (12.4)	13 (29.6)	
Chemotherapy response (RECIST)				**0.004**
Complete	1 (0.5)	1 (0.6)	0	
Partial	196 (95.6)	157 (97.5)	39 (88.6)	
Stable disease	8 (3.9)	3 (1.9)	5 (11.4)	
PI at IDS, median (range)	0 (0–6)	0 (0-6)	0 (0–4)	0.05
Residual disease at IDS				0.72
CC-0	189 (92.2)	149 (92.5)	40 (90.9)	
CC-1	16 (7.8)	12 (7.5)	4 (9.1)	
SCS				**0.01**
Low	64 (31.2)	50 (31.1)	14 (31.8)	
Medium	76 (37.1)	67 (41.6)	9 (20.5)	
High	65 (31.7)	44 (27.3)	21 (47.7)	
Bowel resection				**0.046**
No	120 (58.5)	100 (62.1)	20 (45.5)	
Small-bowel res	8 (3.9)	5 (3.1)	3 (6.8)	
Large-bowel res	65 (31.7)	47 (29.2)	18 (40.9)	
> 1 resection	12 (5.9)	9 (5.6)	3 (6.8)	
Stoma formation				0.62
No	177 (86.3)	140 (87.0)	37 (84.1)	
Yes	28 (13.7)	21 (13.0)	7(15.9)	
Ileostomy	23 (82.1)	18 (85.7)	5 (71.4)	0.39
Colostomy	5 (17.9)	3 (14.3)	2 (28.6)	
Estimated blood loss (ml), median (range)	300 (50–2500)	300 (0–1300)	300 (50–2500)	0.76
Operating time, median (range)^a^	439 (50–840)	432 (240–840)	460 (175–740)	0.23
ICU admission				0.54
No	120 (58.5)	96 (59.6)	24 (54.5)	
Yes	85 (41.5)	65 (40.4)	20 (45.5)	
ICU stay (days), median (range)	1 (1–4)	1 (1–2)	1 (1–4)	0.32
Length of stay (days), median (range)	7 (3–32)	6 (3–32)	7 (4–28)	0.06
Time to start adjuvant chemotherapy, median (range)	36 (25–63)	36 (25–63)	41 (30–60)	0.12
Adjuvant chemotherapy regimen				**< 0.001**
Carboplatinum-paclitaxel	185 (90.2)	153 (95.0)	32 (72.7)	
Other	8 (3.9)	4 (2.5)	4 (9.1)	
No adjuvant	12 (5.9)	4 (2.5)	8 (18.2)	
ACT cycles, median (range), median (range)	3 (1–4)	3 (1-4)	2 (1-3)	**< 0.001**
Maintenance therapy				0.85
Yes	168 (81.9)	132 (82.0)	36 (81.8)	
No	35 (17.1)	27 (16.8)	8 (18.2)	
Missing	2 (1.0)	2 (1.2)	0	
Type of maintenance				
Bevacizumab	60 (29.3)	40 (24.8)	20 (45.5)	**0.008**
PARPi	109 (53.2)	92 (57.1)	17 (38.6)	**0.03**
Creatinine serum level Day 3, median (range)	0.60 (0.04–1.15)	0.59 (0.04–1.15)	0.63 (0.40–1.00)	0.06

*BMI* Body mass index; *PIV* Predictive index value; *NACT* Neoadjuvant chemotherapy; *IDS* Interval debulking surgery; *CC* Completeness of cytoreduction score; *SCS* Surgical complexity score; *ICU* Intensive care unit; *ACT* Adjuvant chemotherapy

^a^Including HIPEC perfusion time

In the subgroup analysis, no significant differences were found on the main patients’ and disease characteristics between FIGO Stage III and FIGO Stage IV patients and between ≤ 4 cycles versus > 4 cycles of NACT. However, exceptions were observed in terms chemotherapy response according to RECIST criteria, being the rate of stable disease after NACT more frequent in patients submitted to > 4 cycles of NACT (11.4 vs. 1.9%; *p* = 0.004).^6^ Also, mean SCS at the time of IDS appeared to be different; patients who received up to 6 cycles of NACT had a higher rate of SCS of 3 compared with women treated with 3–4 cycles of NACT (47.7% vs. 27.3, respectively; *p* = 0.01). Ultimately, a significantly higher rate of both small and large bowel resection was observed in women undergoing delayed IDS compared with those receiving up to 4 cycles of NACT (6.8 vs. 3.1%, 40.9 vs. 29.2 % respectively; *p* = 0.046).

Similarly, in terms of perioperative variables (Tables 1 and 2), no significant differences were detected in the analyzed populations. Of note and despite not significant, a longer median length of inpatient stay (*p* = 0.06) and a higher median Day 3 creatinine serum level (*p* = 0.06) was detected in patients receiving up to 6 cycles of NACT.

Most of our patients received manteinance therapy (81.9%), irrespectively form stage and number of cycles. Regarding the type of manteinance, more patients in the > 4 cycles of NACT group were treated with Bevacizumab (45.5 vs. 24.8%; *p* = 0.008) whilst PARPi were more frequently administered in women receiving ≤ 4 cycles of NACT (57.1 vs. 38.6%; *p* = 0.03). No differences were observed between FIGO stage III and IV.^6^

Overall, the rate of intraoperative complications was quite low (7.8%), and consisted of four diaphragmatic opening during peritonectomy, one liver surface injury, one splenic capsule rupture requiring subsequent splenectomy, one suprahepatic artery injury, one bladder lesion repaired with double-layer suture, and six small-/large-bowel injuries requiring suturing. No differences were found according to stage and number of cycles (Table 3).

**Table 3:** Intra- and postoperative complications

	All cases (*n* = 205)	Figo stage III (*n* = 129)	Figo stage IV (*n* = 76)	*p*	3–4 cycles NACT* (*n* = 161)	> 4 cycles NACT* (*n* = 44)	*p*
Intraoperative complications				0.30			0.78
Yes	16 (7.8)	12 (9.3)	4 (5.3)		13 (8.1)	3 (6.8)	
No	189 (92.2)	117 (90.7)	72 (94.7)		148 (91.9)	41 (93.2)	
Postoperative complications (maximum grade for patients)				0.08			0.33
No	78 (38.0)	43 (33.3)	35 (46.0)		62 (38.5)	16 (36.4)	
G1	74 (36.1)	44 (34.1)	30 (39.5)		58 (36.0)	16 (36.4)	
G2	26 (12.7)	19 (14.7)	7 (9.2)		22 (13.7)	4 (9.0)	
G3	20 (9.8)	16 (12.4)	4 (5.3)		12 (7.5)	8 (18.2)	
G4	0	0	0		0	0	
G5	2 (1.0)	2 (1.6)	0		2 (1.2)	0	
Unknown	5 (2.4)	5 (3.9)	0		5 (3.1)	0	
Patients with at least one G3–G5 postoperative complications at 30 days No. %	22 (11.2)	18 (14.0)	4 (5.3)	0.052	14(8.7)	8(18.2)	0.07
Anastomotic leak	3 (1.5)	2 (1.6)	1 (1.3)		2 (1.2)	1 (2.3)	
Wound dehiscence	4 (2.4)	3 (2.3)	1 (1.3)		2 (1.2)	2 (4.5)	
Abdominal collection	2 (1.0)	2 (1.6)	0		0	2 (4.5)	
Pleural effusion	10 (5.8)	8 (6.2)	2 (2.6)		6 (3.7)	4 (9.1)	
Anemia requiring transfusion	3 (1.5)	3 (2.3)	0		3 (1.9)	0	
Melena	1 (0.5)	1 (0.8)	0		0	1 (2.3)	
Cerebral edema (hyponatraemia)	1 (0.5)	1 (0.8)	0		1 (0.6)	0	

Data are numbers with percentages in parentheses unless otherwise specified

*NACT* Neoadjuvant chemotherapy

The rate of patients reporting severe (G3–G5) postoperative complications within 30 days from surgery was 11.2%. When analyzed according to FIGO stage, we found a higher rate of severe postoperative complications in FIGO stage III than in FIGO stage IV patients, despite not significant (14.0 vs. 5.3%; *p* = 0.052).^6^ Similarly, the subanalysis of severe (G3–G5) 30 days postoperative complication rate according to the number of NACT cycles not reached a statistical significance (8.7 vs. 18.2%; *p* = 0.07; Table 3).

Univariate analysis on risk factors for overall and severe postoperative complications (within 30 days from surgery) showed that having received any kind of bowel resections significantly increased the risk of both any grade and G3–G5 adverse events (odds ratio [OR] 3.54 (1.89–6.63): *p* < 0.001 vs. OR 5.63 (1.99–15.97); *p* < 0.001; respectively). On the contrary, a low/medium SCS appeared to be protective factors for any grade and severe postoperative complications (OR 0.20 (0.09–0.44); *p* < 0.001 vs. OR 0.27 (0.10–0.75); *p* = 0.012; respectively). Of note, the protective role of low SCS and the increased risk for complication of any kind in case of bowel resections performance also was identified in the multivariable model, despite not reaching statistical significance (OR 0.38 (0.13–1.10); *p* = 0.07 vs. OR 2.18 (0.90–5.28); *p* = 0.08).

Ultimatley, being diagnosed with stage [6] IV disease appeared to be a protective factor for 30 days G3–G5 complications development (OR 0.33 (0.11–1.01); *p* = 0.051) at univariate analysis and was confirmed to be the only factor independently associated with a decreased severe complication rate in the multivariable model (OR 0.24 (0.07–0.80); *p* = 0.02; Table 4).

**Table 4:** Univariate and multivariate analysis for overall and G3–G5 complication rate at 30 days from surgery in relation to number of NACT cycles and FIGO stage

	Any grade toxicity		G3–G5 toxicity	
	Univariate OR (95% CI)	Multivariable OR (95% CI)	Univariate OR (95% CI)	Multivariable OR (95% CI)
No. cycles				
3–4	Ref.	Ref.	Ref.	Ref.
> 4 cycles	1.15 (0.58–2.31);* p* = 0.68	1.09 (0.51–2.33); *p* = 0.82	2.25 (0.88–5.78);* p* = 0.07	2.40 (0.78–7.34); *p* = 0.13
FIGO stage				
III	Ref.	Ref.	Ref.	Ref.
IV	0.62 (0.35–1.11);* p* = 0.11	0.56 (0.30–1.06);* p* = 0.07	0.33 (0.11–1.01);* p* = 0.051	0.24 (0.07–0.80); ***p* = 0.02**
Age (years)	1.00 (0.97-1.04); *p* = 0.89	1.00 (0.97–1.04);* p* = 0.99	1.00 (0.95–1.05);* p* = 0.98	1.00 (0.95–1.06); *p* = 0.97
SCS				
Low	0.20 (0.09–0.44); ***p* < 0.001**	0.38 (0.13–1.10);* p* = 0.07	1.0 (N.E.)	0.0 (N.E.)
Medium	0.54 (0.26–1.15);* p* = 0.11	0.86 (0.34–2.19);* p* = 0.75	0.27 (0.10–0.75); ***p* = 0.012**	0.38 (0.11–1.37); *p* = 0.14
High	Ref.	Ref.	Ref.	Ref.
Bowel resection				
No	Ref.	Ref.	Ref.	Ref.
Yes	3.54 (1.89–6.63): ***p* < 0.001**	2.18 (0.90–5.28);* p* = 0.08	5.63 (1.99–15.97); ***p* < 0.001**	1.40 (0.37–5.19); *p* = 0.64

*SCS* Surgical complexity score

Overall, in our series only five (2.4%) patients experienced postoperative complications within 90 days from surgery. Three of them required rehospitalization for surgical correction of vaginal vault dehiscence in two cases and for abdominopelvic collection requiring inpatient drainage in the remaining case.

**Fig. 2:**
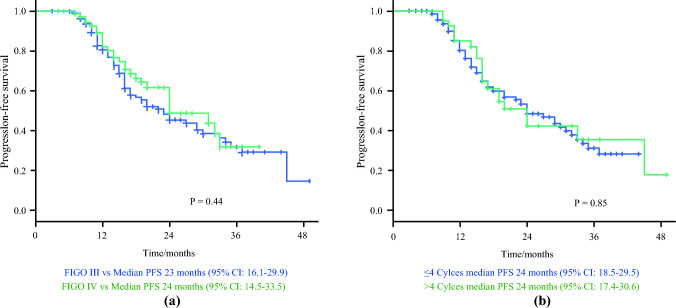
PFS according to stage of disease and number of NACT cycles

With a median follow-up (FU) of 24 months (interquartile range [IQR] 14–34), 90 (43.7%) patients recurred with a 2-year PFS of 47.1% (95% confidence interval [CI] 38.7–55.5). The median PFS in the overall population was 24.0 months (95% CI 17.8–30.2). No difference in PFS between FIGO stage [6] III and FIGO Stage [6] IV patients was detected (*p* = 0.44). Similarly, superimposable PFS was shown between patients receiving 3–4 cycles with respect to women receiving up to 6 cycles of NACT (*p* = 0.85; Fig. 2a, b).

## Discussion

In our experience, the administration of HIPEC at the time of IDS is associated with an overall rate of 11.2% severe postoperative complications, which is significantly lower than what reported in Vandriel’s trial (27%).^3^ Patients receiving up to 6 cycles of NACT and with FIGO stage IV disease had superimposable intraoperative and postoperative complications rate compared with women treated with 3–4 cycles of NACT and/or with FIGO stage III disease.^6^ However, an almost significant increased number of overall and severe postoperative complications was identified in FIGO stage III with respect to FIGO stage IV patients.^6^ Similarly, a trend toward a significant increased number of G3–G5 postoperative complications was detected in women receiving > 4 cycles of NACT. In this context, as patients receiving more cycles of chemotherapy frequently undergo a more aggressive surgery, a multivariate analysis was undertaken in order to clarify any interaction among variables identified as responsible for postoperative complications (i.e., surgical complexity score, bowel resection, age, FIGO stage and number of NACT cycles).^6^ Indeed, only FIGO stage III remained an independent risk factor for severe postoperative complications at multivariate analysis.^6^ In terms of survival outcomes, our study showed comparable PFS between the analyzed populations.

A possible explanation of the different overall and severe morbidity rate of FIGO stage III compared to FIGO stage IV patients undergoing IDS+ HIPEC can be related to a selection bias.^6^ In other words, stage IV women selected for IDS are those showing an exceptional response to NACT, having either a complete disappearance of their metastatic sites or such a reduction in their volume that made surgical resection feasible. Also, the similar good response in the rest of the abdomen made all other variables not significant anymore in the multivariate analysis.

Neither FIGO stage at diagnosis nor number of received NACT cycles appeared to affect perioperative outcomes, even if women submitted to > 4 cycles of NACT showed a trend toward a more deranged postoperative creatinine serum level and longer median hospital stay, which can be explained by (i) the higher rate of small/large bowel resection perfomed in this popualtion, (ii) by the cumulative toxicity from chemotherapy, and/or (iii) by the usual higher rate of comorbidities and disease burden among women scheduled for prolonged NACT.^6,15,16^

Overall, data on the oncological benefit of prolonging NACT are still controversial as no results from RCTs are available yet.^17–21^ Indeed, in real life, a higher number of NACT cycles is usually associated with poor response to platinum and therefore poor outcome. However, data from a recent study on this topic showed that receiving ≥ 4 cycles of NACT could determine an increased response rate with no detrimental effect on both PFS and OS.^17^ Another retrospective multicenter study of more than 300 patients undergoing IDS after a median of 6 NACT cycles has shown positive survival results in patients with NGR at IDS, being median PFS and OS of 19.5 and 49.2 months and 14.8 and 33.0 months in patients with complete and incomplete resection, respectively (*p* = 0.001).^19^

On this background, feasibility of the association of IDS/HIPEC after 6 cycles of NACT has been demonstrated by Marrelli et al.,^22^ who showed limited postoperative morbidity (grade 3 28.2%, including chemotherapy-related toxicity, and no Grade 4–5). They also reported a median PFS of 23 months (95% CI 19–27 months), which is in line with our data.

As we demonstrated that administering up to 6 NACT cycles had no impact on PFS, we can speculate that the combination of prolonged chemotherapy and HIPEC may be able to mitigate the less favorable prognosis of patients who experience delayed IDS, which normally happens in case of either high tumor load or patient’s poor performance status.^22^ Another possible explanation may be that the achievement of (almost) complete resection at the end of the procedure provides a survival advantage in any of the above-discussed treatment pathways.^19,22^

Similar considerations can be made in relation to the superimposable PFS outcomes of FIGO stage III and stage IV patients submitted to HIPEC and IDS.^6^ Even though FIGO stage IV disease appears to be a heterogenous entity in relation to site and number of metastatic lesions which heavily influence treatment strategy, the overall worst prognosis of those patients compared with women with FIGO stage III disease is well established^6,23^ being 5-year OS in this patients less than 20% in most series.^24–27^ Although recent prospective data demonstrated that HIPEC seems to upgrade the prognosis of FIGO stage IV patients, it has to be highlighted that in the present study we are limiting the analysis to a group of hyperselected platinum sensitive cases, in which the achievement of minimal (or zero) residual disease at IDS was possible with or without surgical resection at metastatic sites.^6,28^ Of note, median PFS of our population appeared to be approximately 10 months longer than reported by Vandriel et al.^3^ (24.0 months vs. 14.2 months); whether this may be related to the administration of maintenance therapy in 81.2% of our population is a concrete possibility.

The main drawbacks of our study are represented by the limited inclusion timeframe and the shortness of FUP, so that we are still not able to assess whether the results are confirmed for OS. Moreover, selection biases in the analyzed population cannot bring to a generalization of this approach.

On the contrary, to our knowledge, this is the first series to analyze the effect of HIPEC at the time of IDS in relation to FIGO stage [6] at diagnosis and number of NACT cycles, which goes slightly beyond data coming from currently available RCT.^3,6^ Other strengths of the study are represented by the prospective collection of data, the monocentric nature of the study, the homogeneous treatment setting and HIPEC protocol throughout the whole population.

## Conclusions

A further step on this subject could be represented by the prospective evaluation of HIPEC efficacy in relation to tumor’s molecular pattern together with its potential synergistic effect with currently available target therapies for AOC patients. In relation to the demonstrated absence of additional perioperative complications and possible positive survival outcomes, with this study we offer a background to consider the use of HIPEC with cisplatin 100 mg/m^2^ in selected FIGO stage IV patients and women receiving up to 6 cycles of NACT.^6^
